# Fully digital PET is unaffected by any deterioration in TOF resolution and TOF image quality in the wide range of routine PET count rates

**DOI:** 10.1186/s40658-020-00344-5

**Published:** 2021-01-06

**Authors:** Julien Salvadori, Freddy Odille, Gilles Karcher, Pierre-Yves Marie, Laetitia Imbert

**Affiliations:** 1 Department of Nuclear Medicine and Nancyclotep Molecular Imaging Platform, CHRU-Nancy, Université de Lorraine, F54000 Nancy, France; 2 Université de Lorraine, INSERM, UMR 1254, F54000 Nancy, France; 3 Université de Lorraine, INSERM, UMR 1116, F54000 Nancy, France

**Keywords:** Digital PET, Count rate, Image quality, Time-of-flight

## Abstract

**Purpose:**

Digital PET involving silicon photomultipliers (SiPM) provides an enhanced time-of-flight (TOF) resolution as compared with photomultiplier (PMT)-based PET, but also a better prevention of the count-related rises in dead time and pile-up effects mainly due to smaller trigger domains (i.e., the detection surfaces associated with each trigger circuit). This study aimed to determine whether this latter property could help prevent against deteriorations in TOF resolution and TOF image quality in the wide range of PET count rates documented in clinical routine.

**Methods:**

Variations, according to count rates, in timing resolution and in TOF-related enhancement of the quality of phantom images were compared between the first fully digital PET (Vereos) and a PMT-based PET (Ingenuity). Single-count rate values were additionally extracted from the list-mode data of routine analog- and digital-PET exams at each 500-ms interval, in order to determine the ranges of routine PET count rates.

**Results:**

Routine PET count rates were lower for the Vereos than for the Ingenuity. For Ingenuity, the upper limits were estimated at approximately 21.7 and 33.2 Mcps after injection of respectively 3 and 5 MBq.kg^-1^ of current ^18^F-labeled tracers. At 5.8 Mcps, corresponding to the lower limit of the routine count rates documented with the Ingenuity, timing resolutions provided by the scatter phantom were 326 and 621 ps for Vereos and Ingenuity, respectively. At higher count rates, timing resolution was remarkably stable for Vereos but exhibited a progressive deterioration for Ingenuity, respectively reaching 732 and 847 ps at the upper limits of 21.7 and 33.2 Mcps. The averaged TOF-related gain in signal/noise ratio was stable at approximately 2 for Vereos but decreased from 1.36 at 5.8 Mcps to 1.14 and 1.00 at respectively 21.7 and 33.2 Mcps for Ingenuity.

**Conclusion:**

Contrary to the Ingenuity PMT-based PET, the Vereos fully digital PET is unaffected by any deterioration in TOF resolution and consequently, in the quality of TOF images, in the wide range of routine PET count rates. This advantage is even more striking with higher count-rates for which the preferential use of digital PET should be further recommended (i.e., dynamic PET recording, higher injected activities).

**Supplementary Information:**

The online version contains supplementary material available at 10.1186/s40658-020-00344-5.

## Introduction

Three PET/CT systems using silicon photomultipliers (SiPM) are currently commercialized, namely the Philips Vereos^TM^, the Siemens Biograph Vision^TM^, and the GE Discovery^TM^ MI. Their performances according to the National Electrical Manufacturers Association (NEMA) NU standard have already been described [1–4]. When compared with PMT-based PET, they provide better quality time-of-flight (TOF) images [5–10], leading to enhanced diagnostic confidence and accuracy for oncologic diseases [11–14].

To date, clinical PET/CT systems using SiPM provide the best TOF resolution—i.e., ~ 210 ps for the Biograph Vision^TM^ [2], ~ 310 ps for the Vereos^TM^ [15] and ~ 376 ps for the Discovery MI^TM^ [4]. However, TOF resolution may also be enhanced by further optimizations of PMT-based detectors, as evidenced by the 435 ps TOF resolution reached by the new PoleStarTM m660 PET/CT system (SinoUnion) [16].

The better quality of TOF images is mainly due to a better localization of the emission points, thereby leading to a faster convergence of the iterative reconstruction process and thus to a better tradeoff between contrast and noise [17, 18]. The TOF resolution is the key parameter in this setting and is currently measured at rather low counting rates, around 2 Mcps, as part of the daily procedures of quality control of PET cameras [19], whereas higher counting rates are typically reached in clinical routine [2].

While both Siemens and GE PET/CT scanners use analog SiPMs (aSiPM), the Vereos PET/CT use a detection system based on digital SiPMs (dSiPM) with the particularity that each Single Photon Avalanche Diode (SPAD) is connected to its own readout electronics [20, 21]. Currently, aSiPM and dSiPM have comparable performances with regard to TOF resolution [22], although the latter is expected to better prevent electronic- and detector-related noise [23–26]. When compared to aSiPMs, dSiPMs have active quenching/recharge mechanisms, a property that usually leads to enhanced count rate stability and shortened dead times [23]. In addition, only dSiPMs provide the timestamp of each detected photon and allow providing a full digital processing of all available information.

Moreover, the small aSiPM or dSiPM can be implanted in much higher numbers than the large PMT, this being particularly the case for the Philips Vereos system for which the dSiPMs are individually coupled with scintillator crystals. This leads to a reduction in the number of detected pulses per photodetector and per trigger circuit, thereby preventing count-related rises in dead time and pile-up effect [1, 15]. This feature is the main reason why the Vereos provides a peak noise equivalent count rate (NECR) which is 50% higher and is reached at a 2-fold higher activity concentration, when compared with other systems involving comparable axial field-of-views [1, 27, 28]. Although the count rates achieved during routine PET exams are lower than those corresponding to NECR peaks, small rises in dead time and in the rates of pile-up effects have already been documented at rather low levels of recorded activity on analog PET [29]. However, it is not known whether such small rises may affect the TOF image quality and TOF resolution in the range of routine PET count rates, and whether they may be definitely prevented by the SiPM systems and trigger circuits of digital PET cameras.

The present study is aimed at comparatively assessing the changes in TOF resolution and in the TOF-related enhancement in the image quality, according to the levels of recorded activity and counting rates, between the Vereos fully digital PET camera and the Ingenuity analog PET camera (Philips), with special focus on their respective behaviors in the high range of routine PET count rates.

## Materials and methods

The Vereos and Ingenuity PET-CT systems have already been described in detail elsewhere [15, 29].

### Count-rate performances and count-rate impact on TOF and energy resolutions

The experimental setup was that of the NEMA standard count-rate test. A linear ^18^F source was inserted into the NEMA scatter phantom [30] at the saturating concentrations of 2700 MBq for Vereos PET and of 1700 MBq for Ingenuity PET. This phantom was placed at the FOV center of each camera, but with the line source being off-centered at a radial distance of 45 mm from the central axis. Thereafter, 30 consecutive PET recordings were obtained during a 16-h period and with increasing recording times in order to compensate for radioactive decay. The recordings obtained with an activity higher than that associated with the peak single-count rate were excluded for this analysis.

#### Count-rate performances, NECR, and scatter fraction

**Fig. 1:**
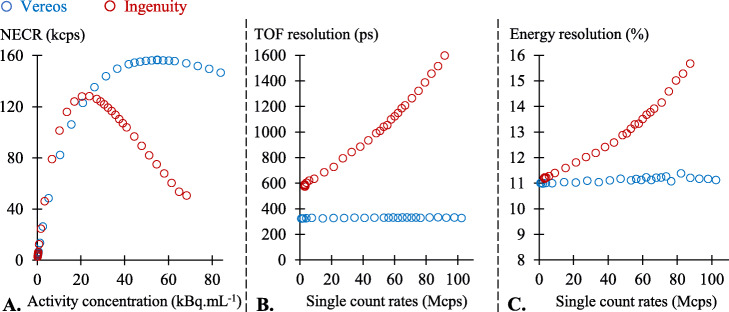
Results obtained from the NEMA count-rate test for the Ingenuity (red) and the Vereos (blue) cameras, with the relationships between noise equivalent count rates and activity concentration (NECR) (**a**) and with the relationships between TOF (**b**) or energy (**c**) resolutions and single-count rates

For each recording, true (T), random (R), and scatter (S) count rates were determined according to NEMA standards [30]. The noise equivalent count rate (NECR), which can be interpreted as the true coincidence rate of an ideal system, namely that which would yield the same signal-to-noise ratio as a real system whose measurement is degraded by scattered and random coincidences, was computed with a noiseless estimate of randoms [31] as follows:

1 $$ \mathrm{NECR}=\frac{T^2}{T+S+R}\kern1.5em $$

where *R* was estimated using delayed coincidence measurements and a variance reduction method [32, 33] (Fig. 1a).

#### Single-count rate/activity relationship

In addition to the coincidence information, the list-mode data of both cameras comprise the prompt, delayed, and single-event rates recorded at each 500 ms interval. Single-count rates, listed at each 500 ms interval, were averaged herein for each of the consecutive recordings.

**Fig. 2:**
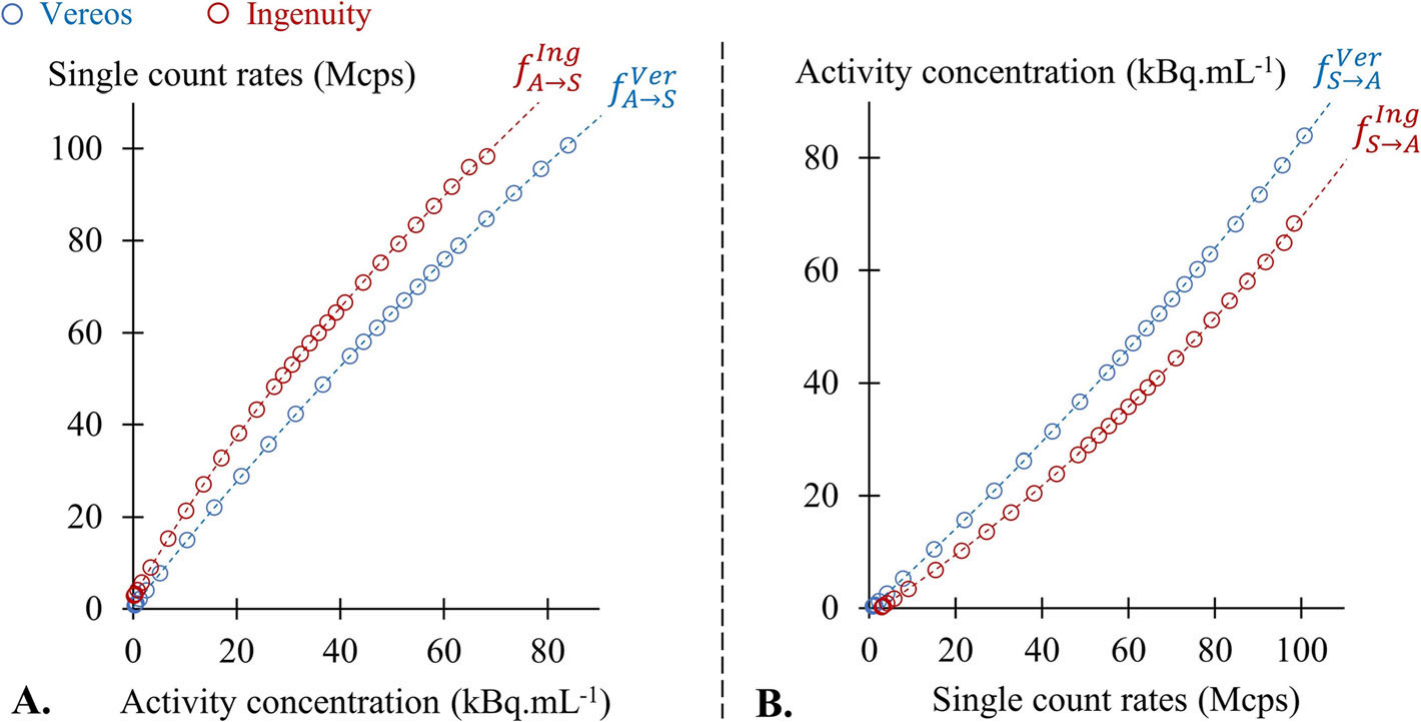
Relationships between single-count rates and activity concentration obtained from the NEMA count-rate test with the Ingenuity (red) and the Vereos (blue) cameras, with the double-exponential regressions predicting: **a** Single-count rates based on activity concentration (${f}_{A\to S}^{Ing}$ and ${f}_{A\to S}^{Ver}$ for respectively Ingenuity and Vereos) and **b** Activity concentration based on single-count rates (${f}_{S\to A}^{Ing}$ and ${f}_{S\to A}^{Ver}$ for respectively Ingenuity and Vereos)

Double-exponential functions were used to fit the relationship between single-count rates (*S*) and activity concentrations (*A*), as well as the reverse relationship between activity concentrations and single-count rates:

2 $$ {f}_{S\to A}(S)\  or\ {f}_{A\to S}(A)=a.{e}^{b.\left(S\  or\ A\right)}+c.{e}^{d.\left(S\  or\ A\right)} $$

where *a*, *b*, *c*, and *d* are the coefficients providing the best fit (all *R*^2^ > 0.9999) for each camera and for each of the 2 functions (.i.e., the function *f*_*S* → *A*_ predicting A from S values and the function *f*_*A* → *S*_ predicting S from A values) (Fig. 2). Corresponding *R*^2^ coefficients and goodness-of-fit criteria are given in Table 1.

**Table 1:** Parameters of the double-exponential function used to model the relationship between single-count rates *S* and activity concentration *A* = *f*_*S* → *A*_(*S*), and the inverse relationship between activity concentration *A* and single-count rates *S* = *f*_*A* → *S*_(*A*), both for the ingenuity (*f*^Ing^) and Vereos (*f*^Ver^) cameras. Two goodness-of-fit criteria are additionally provided for each regression: the coefficients of determination (*R*^2^) and the root mean square error (RMSE)

		Exponential regression parameters *y* = *ae*^*bx*^ + *ce*^*dx*^				Goodness of fit	
		*a*	*b*	*c*	*d*	*R*^2^	*RMSE*
***S =*** *f*_*A* → *S*_(***A***)	${f}_{A\to S}^{Ing}$	76.42	0.006298	− 73.95	− 0.020330	1.0000	0.22910
	${f}_{A\to S}^{Ver}$	183.6	0.001547	− 183.2	− 0.006269	1.0000	0.07143
***A =*** *f*_*S* → *A*_(***S***)	${f}_{S\to A}^{Ing}$	368.9	0.004056	− 370.2	0.002680	0.9999	0.22390
	${f}_{S\to A}^{Ver}$	− 117.9	− 0.001315	117.6	0.004609	1.0000	0.06627

#### TOF and energy resolutions

TOF resolution was determined for each of the consecutive recordings according to a method described in the NEMA NU2-2018 standard [30], based on a previous work by Wang et al. [34], considering the nearest line-of-response points from the linear source as the annihilation points of positrons (Fig. 1b). Briefly, for all true coincidences extracted from the list-mode data, the distances between these annihilation points and the corresponding points computed through TOF differences are considered to correspond to TOF errors (∆t) with the latter represented in a histogram. TOF resolution is then computed as the full width at half maximum (FWHM) of this histogram. According to the NEMA NU2-2018 standard, true coincidences were separated from random and scatter on the basis of a distance from the line source of less than 20 mm [30].

Energy resolution was derived for each of the consecutive recordings from the FWHM value of an energy histogram, the latter being computed from the photon energy of all true coincidences extracted from the same list-mode data (Fig. 1c).

### Extraction and characterization of routine PET count rates

Single-count rates, which are given at each 500 ms interval in the list-mode of both cameras, were extracted from (i) routine PET exams previously recorded on the Vereos or Ingenuity camera after the injection of 3 MBq.kg^-1^ of a ^18^F-labeled tracer (^18^F-FDG, ^18^F-Choline, or ^18^F-DOPA) and (ii) a cardiac exam recorded with a Vereos camera after the injection of 7 MBq.kg^-1^ of Rubidum-82 at stress. The number of selected patients as well as the number of collected samples of single rates are reported in Table 2 and this, for each tracer type and for each camera. As seen in Table 2, the main patient characteristics were comparable between patients imaged with the Vereos and those imaged with the Ingenuity camera.

**Table 2:** General patient characteristics and main PET parameters recorded or computed in the different groups imaged in clinical routine with the Vereos (digital-PET) or Ingenuity (analog-PET) camera and after the injection of 3 MBq.kg^-1^ of current ^18^F-labeled tracers, with an additional patient imaged after the injection of 7 MBq.kg^-1^ of ^82^Rb on the Vereos camera. Continuous variables are presented with median values [minimum – maximum values after exclusion of outliers]

	Ingenuity TF			Vereos			
**Tracer type**	^18^F-FDG	^18^F-Choline	^18^F-DOPA	^18^F-FDG	^18^F-Choline	^18^F-DOPA	^82^Rb
**Number of patients**	10	10	1	10	10	1	1
**Gender**	Female	Male	Female	Female	Male	Female	Male
**Age (years)**	76 [67-82]	70 [67-75]	72	71 [71-77]	72 [70-76]	65	55
**Body weight (kg)**	68 [65-69]	78 [74-84]	84	63 [59-69]	80 [77-81]	90	159
**Injection-to-recording delay-time (min)**	69 [65-72]	14 [12-17]	0	67 [65-74]	24 [17-25]	0	0
**Recorded area**	Whole body	Whole body	Pelvis	Whole body	Whole body	Pelvis	Thorax
**Number of list-mode samples**	18534	20757	2382	24814	21210	2401	961
**Single count rate** **(Mcps)**	8.1 [5.8-10]	14.2 [7.4-21.7]	19.2 [17.7-20.1]	4.6 [2.3-6.0]	7.6 [3.7-12.6]	10.5 [7.9-13.3]	4.6 [0.5 29.5]
**Corresponding activity concentration on NEMA phantom (kBq.mL**^**-1**^**)**	2.9 [1.7-3.9]	6.2 [2.5-10.4]	9.0 [8.1-9.5]	2.9 [1.3-3.9]	5.1 [2.3-8.7]	7.2 [5.3-9.2]	3.0 [0.3-20.8]
**Corresponding TOF resolution (ps)**	631 [621-643]	677 [628-732]	713 [702-719]	328 [325-332]			
**Corresponding energy resolution (%)**	11.36 [11.28-11.43]	11.6 [11.34-11.82]	11.7 [11.68-11.77]	11.01 [10.98-11.05]			

**Fig. 3:**
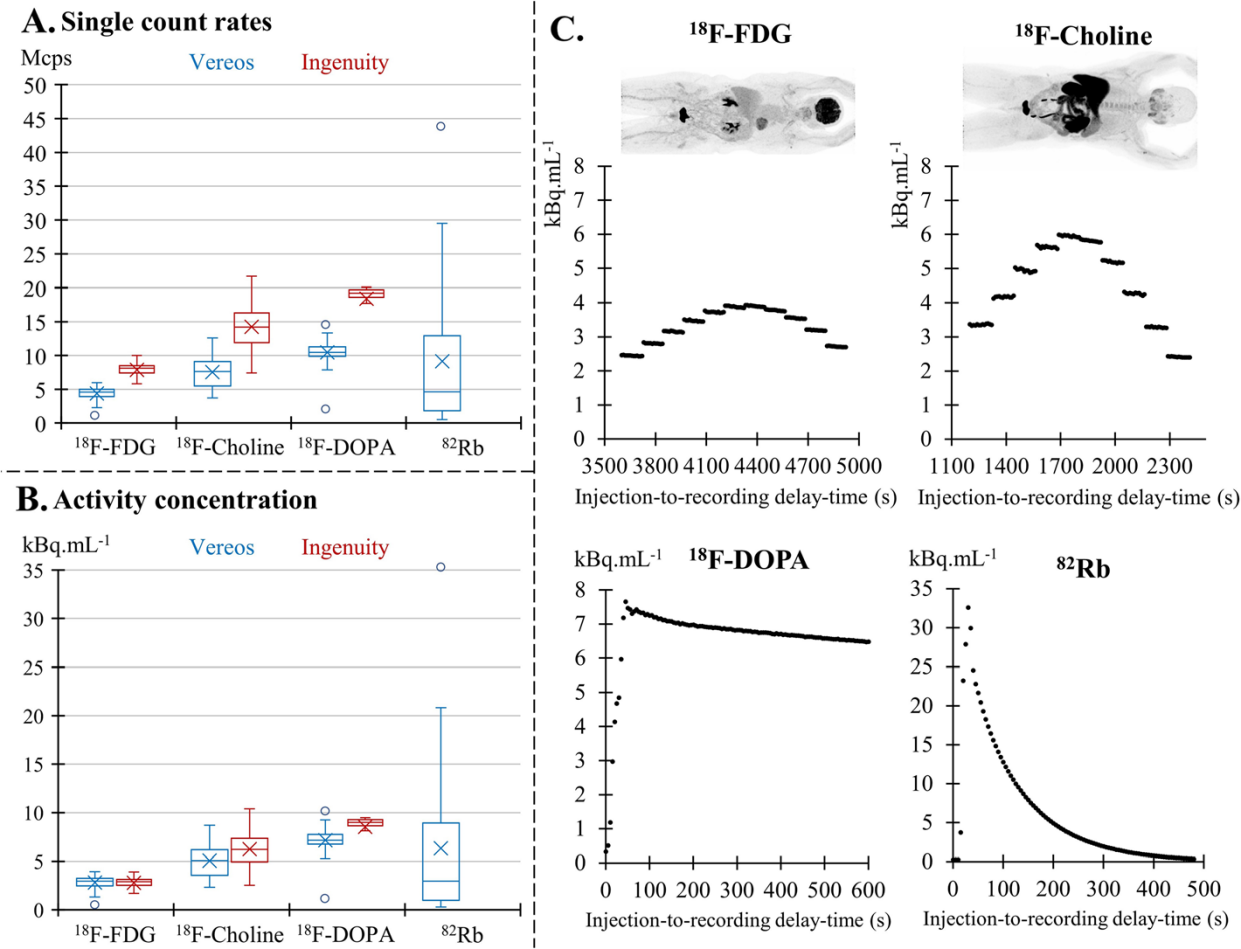
Box-plot representation of **a** distributions of single-count rates collected during routine PET exams as a function of tracer type (^18^F-FDG, ^18^F-Choline, ^18^F-Dopa, Rubidium-82) and camera type (blue for Vereos and red for Ingenuity), **b** corresponding activity concentrations from the NEMA scatter phantom (i.e., obtained through the relationship displayed in Fig. 2a), with **c** examples of time-evolutions of the count rates recorded during whole-body PET exams with ^18^F-FDG and with ^18^F-Choline, and during dynamic exams focused on the pelvis region for ^18^F-DOPA and on the cardiac region for Rubidium-82

A corresponding activity concentration of the NEMA phantom was computed for these single-count rates collected in patients by using the aforementioned function *f*_*S* → *A*_ described above. The distributions of these count rates and of corresponding activity concentrations were analyzed according to tracer type and camera type and while considering all samples of 500 ms interval recorded in all bed positions and in all patients. As depicted in Fig. 3a, b, this distribution is represented through box plots along with the display of median values and interquartile ranges, as well as with the maximal and minimal values after excluding the outliers (i.e., values higher than 1.5-fold the upper limit of the 3rd quartile or lower than 1.5-fold the lower limit of the 1st quartile). These maximal and minimal values of the box plots were deemed to reflect the upper and lower limits for count rate, as well as for activity concentrations.

**Fig. 4:**
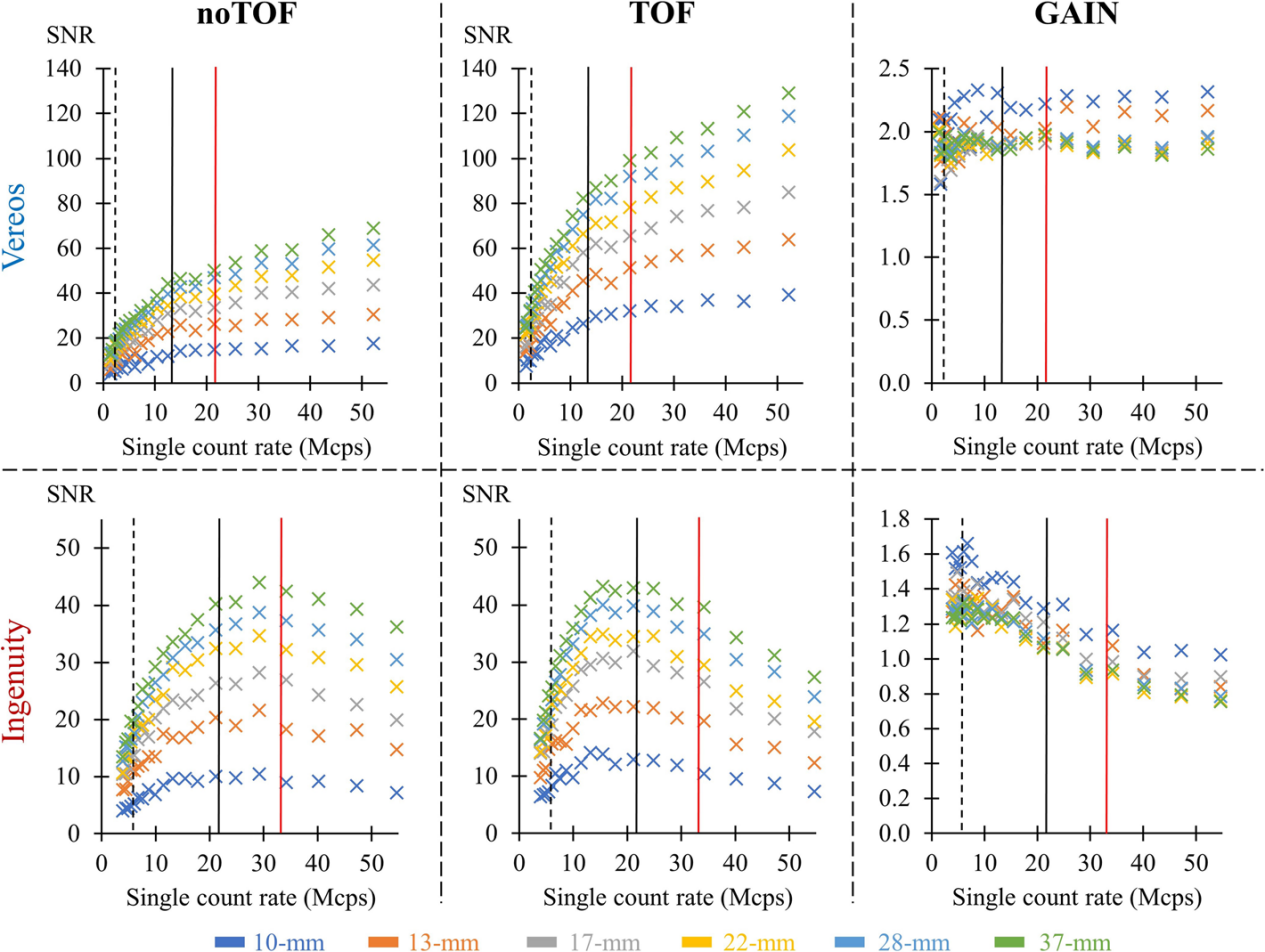
Single-count rates versus signal-to-noise ratios (SNR) of the 10 to 37-mm diameter hot spheres of the IEC phantom for the noTOF (left panels) and TOF (median panels) images from the Vereos (upper panels) and Ingenuity (lower panels) cameras, together with the corresponding TOF-related gain, i.e., the ratio between the SNR of TOF and noTOF images (right panels). The dashed and solid vertical black lines correspond to respectively the lower and upper levels of single-count rates for the PET exams performed with the injections of 3 MBq.kg^-1^. The solid red vertical line corresponds to the estimated upper level of single-count rates for the PET exams performed with the injections of 5 MBq.kg^-1^

### TOF-related enhancement in the image quality in the upper range of routine PET count rates

#### Recording and reconstruction of PET images

The NEMA IEC phantom is an anthropometric body phantom containing 6 spheres with diameters of 10, 13, 17, 22, 28, and 37 mm, as well as a cylindrical central “lung” insert, filled with a low-density material [30].

**Fig. 5:**
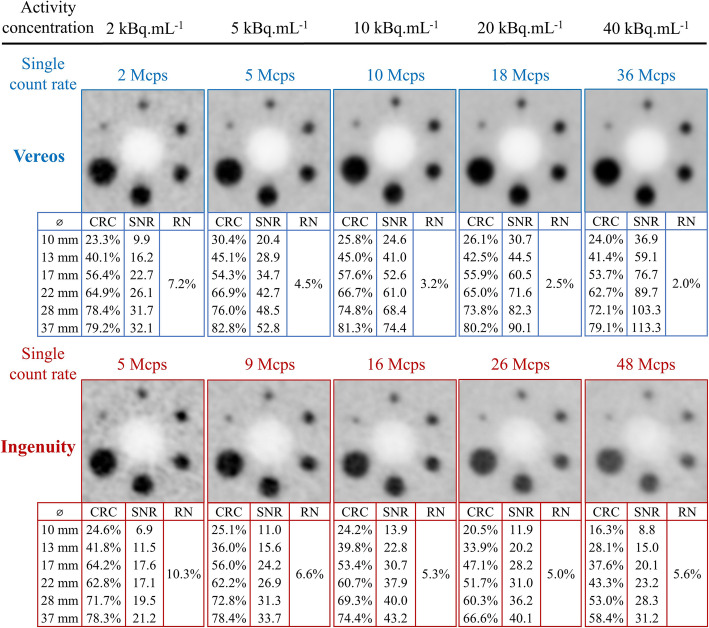
Representative examples of PET slices of the IEC phantom recorded with the TOF information with the Vereos (upper panels) and the Ingenuity (lower panels) at count rates corresponding to the same levels of activity concentrations for both cameras (i.e., at background concentrations of the IEC phantom of 2, 5, 10, 20, and 40 kBq.mL^-1^). The values of signal-to-noise ratio (SNR) and contrast recovery coefficients (CRC) for the 10 to 37-mm diameter hot spheres are inserted in each image, together with the background relative noise (RN)

An IEC phantom, filled with an ^18^F concentration of 60 kBq.mL^-1^ in the background and 4-fold higher concentrations in the 6 spheres, was positioned at the centers of the Vereos and Ingenuity cameras for serial 3 min PET recordings at each 30-min interval, until the background concentration fell under the level of 1 kBq.mL^-1^. Images were reconstructed with and without TOF information (TOF and noTOF images, respectively), with a fixed number of 10 subsets and with the number of OSEM iterations previously shown to maximize the signal-to-noise ratio [35] of the 10-mm sphere irrespective of the level of recorded activity—i.e., 1 and 2 OSEM iterations for TOF images from the Vereos and Ingenuity, respectively, and 4 OSEM iterations for noTOF images from both cameras [9]. No image filter was applied and the relaxation parameter was set to 1.0, the latter controlling the magnitude of the image change induced by each iteration.

The single-count rates, listed at each 500-ms interval, were averaged for each 3-min recording (this 3-min period corresponding to a total of 360 samples).

#### Image quality metrics

Signal-to-noise ratio (SNR) and contrast recovery coefficient (CRC) were computed for each sphere of the IEC phantom and for each of the consecutive PET recordings according to the following formulas [30, 35]:

3 $$ {\mathrm{CRC}}_{\mathrm{sphere}}=\frac{\frac{S_i}{B_i}-1}{\frac{a_H}{a_c}-1}\ast 100 $$

4 $$ {\mathrm{SNR}}_{\mathrm{sphere}}=\frac{S_i-{B}_i}{\sigma_i} $$

where *S*_*i*_ represents the mean voxel activities extracted from a circular 2D region-of-interest (ROI), matching the sphere of *i* diameter and placed on the slice passing through the sphere center; *B*_*i*_ and *σ*_*i*_, respectively represent the mean and standard deviation of the activities from all voxels setting within the 60 background regions-of-interest (ROIs) defined by the NEMA standard for the sphere of *i* diameter; and $\frac{a_H}{a_c}$ is the actual ratio of activity concentrations between spheres and background, which was set at 4 in the present instance.

Background relative noise was computed through the coefficient of variation of the activities from the 60 background 37-mm diameter ROIs, with the resulting values converted in percentages [36]:

5 $$ \mathrm{RN}=\frac{\sigma_{37 mm}}{B_{37 mm}}\ast 100 $$

## Results

### Count-rate performances and count-rate impact on TOF and energy resolutions

As evidenced in Fig. 2, the single-count rates from both cameras were closely linked to the activity concentrations during the NEMA count-rate test. However, this relationship was shifted to higher single-count rate values for the Ingenuity camera, due to its higher absolute count sensitivity [1, 29].

A similar upward shift was documented for the noise equivalent count rate (NECR) of the Ingenuity, as compared with that of the Vereos, but only up to the NECR peak of the Ingenuity, a level where the two curves intersect (Fig. 1a). The NECR peak of the Vereos camera was reached at a greater than 2-fold higher activity than that of the Ingenuity (54 vs. 24 kBq.mL^-1^ (Fig. 1a).

For the large range of single-count rates, tested at up to 100 Mcps, the TOF and energy resolutions of the Vereos were remarkably stable, at approximately 326 ps and 11%, respectively (Fig. 1b, c). For the Ingenuity, by contrast, TOF and energy resolutions exhibited progressive deteriorations according to count rate.

### Extraction and characterization of routine PET count rates

For both cameras, the single-PET count rates collected in patients were higher for ^18^F-choline and ^18^F-DOPA comparatively to ^18^F-FDG (see Fig. 3a), which was mainly explained by much shorter injection-to-recording delay times (Table 2). Single-count rates were even higher at the onset of the Rubidium-82 Vereos PET exam, presumably at the time of the first cardiac pass of the tracer bolus (Fig. 3c).

Due to the aforementioned higher absolute count sensitivity [1, 29], the Ingenuity exhibited higher single-count rates than the Vereos (Fig. 3a). However, this difference was much less pronounced when these count rates were replaced by the corresponding concentrations from the scatter phantom obtained from the relationships displayed in Fig. 2b for each camera (Fig. 3b).

The count rates of all of the PET exams performed with ^18^F-labeled tracers ranged from 2.3 to 13.3 Mcps for the Vereos and from 5.8 to 21.7 Mcps for the Ingenuity, corresponding to the upper and lower limits of the box plots displayed in Fig. 3a.

#### Estimation of top levels of routine PET count rates

For routine protocols performed with injected doses of 5 MBq.kg^-1^ instead of 3 MBq.kg^-1^ of ^18^F-labeled tracers [37], the upper limits were presumptively estimated to be higher, reaching 21.6 Mcps for the Vereos and 33.2 Mcps for the Ingenuity. These estimated count rates values (*S*_estimated_) were obtained from the upper limits of the count rates documented after injection of 3 MBq.kg^-1^ for the Vereos (S = 13.3 Mcps) and Ingenuity (S = 21.7 Mcps) systems with the following equation:

6 $$ {S}_{\mathrm{estimated}}={f}_{A\to S}\left(C.{f}_{S\to A}(S)\right) $$

where *f*_*S* → *A*_ is the double-exponential function predicting the activity concentrations of the NEMA count-rate phantom based on single-count rates for a given camera (Fig. 2b), and *f*_*A* → *S*_ is the double-exponential function predicting the single-count rates of the same camera based on activity concentrations (Fig. 2a). The constant C corresponds to the change in activity concentration by a factor of 5/3, corresponding to the shift in injected dose from 3 to 5 MBq.kg^-1^.

A similar method was applied to estimate an upper limit of 39.5 Mcps for the Rubidium-82 PET exam recorded on the Ingenuity camera, based on the upper limit observed for this exam on the Vereos camera (*S*_Ver_ = 29.5 Mcps. For this purpose, the following equation was used:

7 $$ {S}_{\mathrm{estimated}}^{\mathrm{Ing}}={f}_{A\to S}^{Ing}\left({f}_{S\to A}^{Ver}\left({S}_{\mathrm{Ver}}\right)\right) $$

where ${f}_{S\to A}^{Ver}$ is the double-exponential function predicting activity concentrations based on the single-count rates of the Vereos camera (29.5 Mcps) (Fig. 2b), and ${f}_{A\to S}^{Ing}$is the double-exponential function predicting the single-count rates of the Ingenuity camera based on activity concentration (Fig. 2a).

The validity of these estimations was strengthened by comparisons with actual measurements, although these comparisons could not be obtained with human data (no patient was injected in this instance with 5 MBq.kg^-1^ of tracer and no patient was investigated with Rubidium-92 with the Ingenuity camera) and only on the IEC phantom data obtained on a wide activity range (see Additional file 5: Appendix 1).

### TOF-related enhancement in the image quality in the upper range of routine PET count rates

#### TOF resolution

As mentioned above, the TOF resolution of the Vereos was stable around 326 ps irrespective of the level of recorded count rate (Fig. 1b). By contrast, the TOF resolution of the Ingenuity rose from 621 to 732 ps between 5.8 and 21.7 Mcps, corresponding to the lower and upper limits, respectively, of the count rates documented with the injection of 3 MBq.kg^-1^ of ^18^F-tracers. This TOF resolution of the Ingenuity deteriorated even further at the upper limits of count rates expected to be achieved with the injection of 5 MBq.kg^-1^ of ^18^F-tracers (847 ps at 33.2 Mcps) or of Rubidium-82 (899.5 ps at 39.5 Mcps).

#### TOF-related gain in SNR

The TOF-related gain in SNR was computed through the ratio between the SNR from TOF and noTOF images.

As evidenced in Fig. 4, the TOF-related gain in SNR provided by the Vereos was of 2, on average, and was clearly stable according to single count rates, although slight variations were documented according to sphere size (i.e., this gain was consistently higher for the smallest as opposed to the largest spheres).

For the Ingenuity, the TOF-related gain in SNR exhibited comparable variations according to sphere size, although was found to deteriorate according to count rate. More precisely, the averaged TOF-related gain decreased from 1.36 at the lower limit of routine PET count rate of the Ingenuity (5.8 Mcps), to 1.14 and 1.00 at the upper limit of routine PET count rate documented for this camera with the respective injections of 3 MBq.kg^-1^ and 5 MBq.kg^-1^ (Fig. 4). This gain even fell below 1 (0.90) at the upper limit of the count rate estimated during the Rubidium-82 PET exam with the Ingenuity (Fig. 4).

In addition, as seen in Fig. 4, a saturation of the SNR could be documented for the Ingenuity but not the Vereos camera with SNR peaks of the Ingenuity achieved around 30 Mcps on the noTOF images and even earlier, around 15 Mcps, on TOF images. Consequently, the SNR achieved by TOF images in the upper range of routine PET count rates were dramatically higher for the Vereos than for the Ingenuity. In particular, the Vereos-to-Ingenuity ratio of the averaged SNR of the 6 spheres was 1.3 at the lower limits of the count rates documented in clinical routine (i.e., 2.3 Mcps for Vereos and 5.8 Mcps for Ingenuity). However, this ratio further increased to 2.0 and 2.6 at the upper limits of the count rates documented for each camera at respectively 3 and 5 MBq.kg^-1^ of ^18^F-labeled tracers and to 3.3 at the upper limits corresponding to the Rubidium-82 exams.

Representative examples of TOF PET images of the IEC phantom are given in Fig. 5 for both cameras and for growing count rate conditions.

Finally, the count-related deterioration in the TOF-related gain in SNR from the Ingenuity camera was found to be in line with concomitant deteriorations in the gains of both contrast and noise (see Additional files 1 and 2: Figures S6 and 7).

It may additionally be pointed out that, for the Ingenuity, the TOF-related gain in SNR and in contrast fell below 1 with the rise in count rate (see Fig. 4 and Additional file 1: Figure S6, respectively), yielding evidence of a paradoxical worsening of the quality for images reconstructed with the TOF information.

## Discussion

Fully digital PET was previously shown to significantly improve the quality of TOF images due to an enhanced TOF resolution as compared with analog PET and to better prevent the count-related rises in dead time and pile-up effects due to the use of digital SiPM systems with small trigger domains (i.e., small detection surfaces associated with each trigger circuit). The present study provides evidence that combined together, these two properties are particularly advantageous in the high range of the activities recorded by PET in clinical routine and where digital PET is particularly better suited to prevent the deterioration in TOF resolution and, consequently, in the TOF image quality. Although the majority of our data were obtained with ^18^F-labeled tracers, they may likely be extrapolated to all other PET tracers, depending on the level of single-count rates achieved during recording.

A previous study had already shown that count rate-related deteriorations in TOF and energy resolutions of the Ingenuity PMT-based PET cameras were virtually linear and started at very low count rates [29]. To our knowledge, the present study is the first to show that this deterioration has a significant impact on the image quality, especially in the upper range of routine PET count rates.

The TOF resolution of PET cameras is commonly assessed as part of daily control procedures with a point source of ^22^Na and thus, under low count rate conditions (~ 2 Mcps). In clinical routine, however, the activity recorded by PET is higher and varies according to injected doses as well as patient characteristics such as body weight or renal function. In addition, this recorded activity varies according to tracer type and recording protocols with significant fluctuations during the course of each PET exam. Indeed, as illustrated herein in Fig. 3c, higher activities are generally recorded during the abdominal and pelvic recording steps for whole body recordings of current ^18^F-labeled tracers, as well as during the early recording phases for dynamic PET exams.

In the present study, the levels of single-count rates were able to be collected during the course of current PET exams, at each 500-ms interval, and these levels were put in correspondence with the TOF resolution and image quality metrics measured at the same count rates with the NEMA count-rate phantom and the IEC phantom, respectively. It should be emphasized, however, that the absolute count sensitivity of the Vereos camera is lower than that of the Ingenuity camera [1, 29], due to [1] a lower axial field of view (164 vs. 180 mm) [2], shorter crystals (19 vs. 22 mm) [3], larger inter-pixel dead space, and [4] a smaller energy window (164 vs. 276 keV). This point is clearly evidenced in Fig. 2a where the curves from single-count rates of both cameras are put in correspondence with the activity concentrations from the NEMA count-rate phantom.

The higher count sensitivity of the Ingenuity is further illustrated by the observation in Fig. 3a in which this camera was also associated with higher single-count rates than the Vereos camera during the recording of routine PET exams. However, as evidenced in Fig. 3b, this difference between the 2 cameras was clearly minimized after conversion of these routine count rates into recorded activities. Such equivalence in terms of recorded activities was expected since all study patients were submitted to the same injection protocols and their anthropometric and clinical characteristics were comparable between the two cameras (Table 2). In these conditions, both the body distribution and time-evolution of the tracer activities during the PET exams could indeed be expected to be roughly comparable between the two cameras, even if they were associated with higher absolute count rates with the Ingenuity as opposed to the Vereos camera.

As a result, the upper limits of the routine PET count rates were reached herein with the Ingenuity but not with the Vereos cameras, due to the aforementioned difference in absolute sensitivity. The limit attained was 21.7 for the injection of 3 MBq.kg^-1^ of current ^18^F-labeled tracers and was estimated at 33.2 Mcps for 5 MBq.kg^-1^ of ^18^F-labeled tracers and at 39.5 Mcps for 7 MBq.kg^-1^ of Rubidium-82. At these upper limits, which were all reached with the Ingenuity, both the TOF resolution and the TOF-related gain in SNR were found to be remarkably stable for the Vereos camera throughout the broad range of activities tested herein (Figs. 1b and 4). In contrast, with the Ingenuity camera, both parameters deteriorated progressively in an almost linear relationship according to count rates (see Figs. 1b and 4), and with significant deteriorations being already documented between the lower and upper limits of the count rates observed after the injection of only 3 MBq.kg^-1^ of current ^18^F-labeled tracers, i.e., from 621 ps to 732 ps for TOF resolution and from 1.36 to 1.14 for the TOF-related gain in SNR.

Finally, at the upper limit of the count rates achieved with the injection of 3 MBq.kg^-1^ of ^18^F-labeled tracers (21.7 Mcps), the SNR achieved by the TOF images from the Vereos was, on average, 100% higher than the SNR achieved by the TOF images from the Ingenuity. By contrast, this percentage was only of 30% at the lower limit (5.8 Mcps). This superiority of the SNR achieved by the TOF images of the Vereos was even more pronounced at the upper limits of the count rates likely achieved with the injection of 5 MBq.kg^-1^ of ^18^F-labeled tracers (33.2 Mcps), as well as with the Rubidium-82 protocol (39.5 Mcps), with respective enhancements of 160% and 220%, as compared with the SNR achieved by the TOF images of the Ingenuity camera.

As already stated above, the dSiPM-based detection system of the Vereos camera was already shown to be highly advantageous for its performance at high count rates [1, 15]. This point is well illustrated by the fact that the NECR peak was documented for activity levels which were two-fold higher for the Vereos than for the Ingenuity system. The latter also likely explains the further deteriorations in the image quality of the Ingenuity camera beyond this NECR peak. However, the present study shows that TOF resolution as well as the quality of the TOF images from the Ingenuity deteriorated at much lower count rates than those corresponding to the NECR peak.

This progressive worsening in the quality of the TOF images from the Ingenuity could be directly attributed to the concomitant deterioration in TOF resolution, especially for the count rates setting within the usual routine ranges (i.e., under the 30 Mcps level corresponding to the SNR peak on noTOF images (see Fig. 4)). Indeed, by using a method initially described by Wang et al. [34], the TOF resolution of the Ingenuity was found to deteriorate linearly as a function of counting rates, even under the 30 Mcps level, whereas the TOF resolution of Vereos was very short and stable regardless of the count rate level.

An additional and paradoxical observation was that, at count rates setting over 10 Mcps and 30 Mcps, respectively, the averaged SNR and contrast values from the spheres were lower on TOF than on noTOF images from the Ingenuity (Fig. 4 and Additional file 1: Figure S6). This is likely the result of the fact that the count-related deterioration in TOF resolution may not be accurately taken into account in the reconstruction process of TOF images. This hypothesis is further strengthened by previous studies having shown that the use of an incorrect Gaussian TOF kernel, due to miscalibration or count rate influence, leads to increased noise and to lower lesion contrast [38–40]. Finally, this hypothesis was definitely confirmed through additional “in silico” experiments with Monte-Carlo simulations where significant deteriorations in contrast and SNR were documented for reconstructions performed with misfit TOF kernel values (see Additional file 4: Figure 8). It may be mentioned that not only the width but also the shape of the TOF distribution needs to be modeled accurately. This distribution is commonly considered as Gaussian, although a Laplace distribution could be more appropriate for very high timing resolutions (< 50 ps) [41].

It should be pointed out that not only the TOF resolution but also the energy resolution of the Ingenuity camera was affected at increasing values of recorded count rates (Fig. 1c). In the range of routine count rates, however, the maximal amplitude of the deterioration in energy resolution was only of one percentage point for the Ingenuity, corresponding to the difference between the level of 11.3%, at the lower limit achieved with ^18^F-tracers, and the level of 12.4%, the maximal count rate estimated for ^82^Rb. On additional “in silico” experiments, the impact on the image quality parameters was found to be negligible for this small deterioration in energy resolution compared to that induced by the concomitant deterioration in TOF resolution (results not shown). The simulated camera was the Vereos for which the GATE Monte Carlo model was previously validated by a direct comparison with experimental data [42].

The degradation in TOF resolution with the increase in count rate is a deleterious consequence of the increases in dead time and pile-up effect, two parameters which are inversely linked to the size of trigger domains (i.e., the detection surface associated with each trigger circuit). However, the number of photodetectors is dramatically higher for the Vereos than for the Ingenuity system (23040 dSiPMs vs. 420 PMTs), with a similar difference being observed between the 2 cameras for the number of trigger circuits (5760 vs. 28). Therefore, the Vereos involves a much smaller trigger domain than the Ingenuity (0.64 cm^2^ vs. 132.48 cm^2^), leading to a drastic reduction in the data flow per trigger circuit and thus, to better prevention against dead time and pile-up effects.

As detailed in Additional file 3: Table S3, trigger domains are smaller on the commercially available digital PET systems than on the analog PET systems, although they are far from being equivalent within each of these two categories.

“*In particular, the PMT-based Biograph mCT PET/CT* (*Siemens*) *involves a trigger domain of only 27 cm*^*2*^ (*one domain per detector block), and therefore, its vulnerability to high-count rates may be expected to be much lower than that of the Ingenuity (one domain per detector panel*) [43–45].”

In the present study, we analyzed the behavior of timing resolution (assessed on the NEMA count-rate phantom) and of TOF image quality (assessed on the IEC phantom) in the wide range of routine PET count rates. For this purpose, the level of single-count rates from a given camera was considered to have comparable consequences on the TOF images from phantoms and patients. However, it must be recognized that these consequences could vary according to the spatial distribution of the recorded radioactive sources. It could even be considered that local deteriorations in TOF-image quality might occur in the absence of any evident increases in total count rates, when only a few trigger domains are saturated, due to radioactive sources lying in close proximity.

The count rate distribution between the different trigger domains of a given camera is likely not identical when imaging phantoms or patients, as well as when imaging patients with different anthropometric characteristics or injected with different tracers. These considerations constitute a limitation in the interpretation of our results.

Another limitation was that no PET data were available for measuring the extreme levels of the count rates that may be achieved in clinical routine with these cameras (i.e., after the injection of 5 MBq.kg^-1^ of FDG or during the early vascular phase of an ^82^Rb exam for the Ingenuity). These count rates could only be estimated and the accuracy of these estimations may be questioned, even if we have also observed that our estimation method yields coherent results when applied for estimating the count rates achieved not in patients but on the IEC phantom and on a large range of recorded activity. The relative difference with regard to actual values is ≤ 2% when estimating the count rates achieved at a higher activity than those prescribed in our patients and ≤ 7% when estimating the Ingenuity count rates corresponding to the count rates recorded with the Vereos (see Additional file 5: Appendix 1).

However, all of these limitations likely do not challenge our main observation that, contrary to PMT-based PET with large trigger domains, fully digital PET is unaffected by any deterioration in TOF resolution and the TOF image quality in the wide range of the global PET count rates observed in clinical routine.

Finally, we only used an OSEM image-reconstruction algorithm, but our results would likely be unchanged with other TOF-based algorithms, such as Bayesian penalized likelihood algorithms [46].

In conclusion, fully digital PET was already shown to provide significant advantages, when compared with PMT-based PET, due to an enhanced TOF resolution, as well as to a better prevention of the count-related rises in dead time and pile-up effects. The present study provides new compelling evidence that combined together, these properties are particularly advantageous in the upper range of activities recorded by PET in clinical routine and where digital PET is unaffected by any deterioration in the TOF resolution and TOF image quality, contrary to the Ingenuity PMT-based PET. While this advantage is already significant in the range of routine PET count rates achieved with limited injected doses, it becomes even more prominent with higher count rates for which the preferential use of digital PET should be further recommended (i.e., dynamic PET recording, higher injected activities).

## Supplementary Information

**Additional file 1: Figure S6.** Single count rates versus contrast recovery coefficients (CRC) of the 10 to 37-mm diameter hot spheres of the IEC phantom for the noTOF (left panels) and TOF (median panels) images from the Vereos (upper panels) and Ingenuity (lower panels) cameras, together with the corresponding TOF-related gain, i.e. the ratio between the CRC of TOF and noTOF images (right panels). The vertical lines are defined in the legend of Figure 4.

**Additional file 2: Figure S7.** Single count rates versus background relative noise of the IEC phantom for the noTOF (left panels) and TOF (median panels) images from the Vereos (blue) and Ingenuity (red) cameras, together with the corresponding TOF-related gain, i.e. the ratio between the background relative noise of TOF and noTOF images (right panels). The vertical lines are defined in the legend of Figure 4.

**Additional file 3: Table S3**: Characteristics of currently commercialized PMT- and SiPM-based PET cameras with a special focus on the size of their trigger domains.

**Additional file 4: Figure S8.** Data derived from simulated images of the IEC phantom, obtained with the GATE/Geant4 platform, reconstructed with the CASToR software (OSEM, 2 iterations 10 subsets) and confirming that incorrect TOF-kernel leads to a decrease in the contrast and SNR of TOF images. More precisely, these data show the comparative evolutions of the contrast recovery coefficients (upper panel) and SNR (middle panel) of hot spheres for increasing values of TOF resolution between the TOF images reconstructed with TOF-kernels matching exactly the increasing values of TOF resolution (left panel) and those reconstructed with misfit TOF-kernels kept unchanged at 300 ps (right panel). Simulated PET slices, obtained for various TOF resolutions, are shown in the bottom panels.

**Additional file 5: Appendix 1** IEC phantom assessment of our methodology of count rate estimation in patients

## Data Availability

The datasets used and/or analyzed during the current study are available from the corresponding author on reasonable request.
