# Supplementary figures and images for: Fully digital PET is unaffected by any deterioration in TOF resolution and TOF image quality in the wide range of routine PET count rates
Source: EJNMMI Phys. 2021 Jan 6;8:1. doi: 10.1186/s40658-020-00344-5 (PMC7788141; doi:10.1186/s40658-020-00344-5)

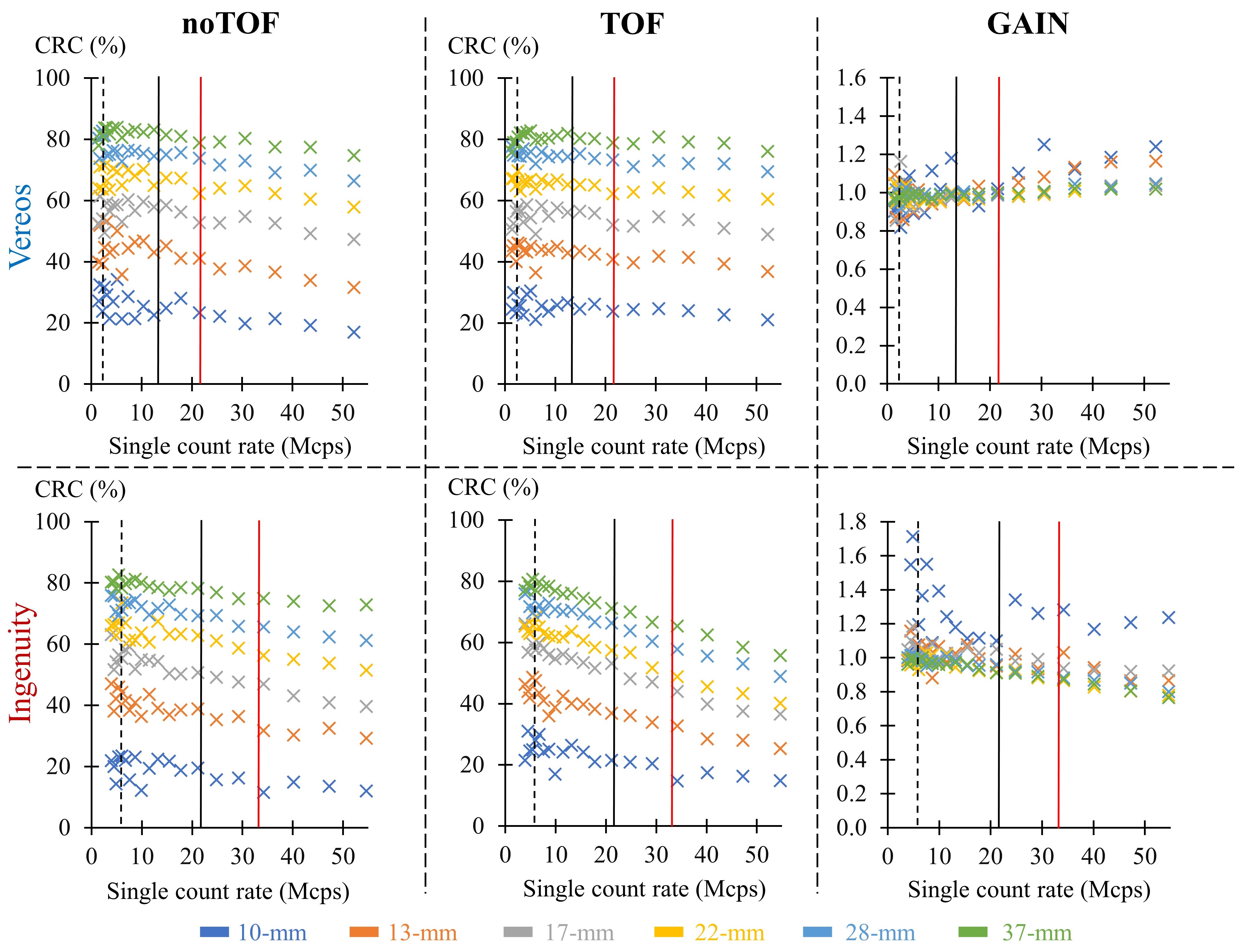

Supplement: Supplementary file 1 — Additional file 1: Figure S6. Single count rates versus contrast recovery coefficients (CRC) of the 10 to 37-mm diameter hot spheres of the IEC phantom for the noTOF (left panels) and TOF (median panels) images from the Vereos (upper panels) and Ingenuity (lower panels) cameras, together with the corresponding TOF-related gain, i.e. the ratio between the CRC of TOF and noTOF images (right panels). The vertical lines are defined in the legend of Figure 4. [file 40658_2020_344_MOESM1_ESM.jpg]

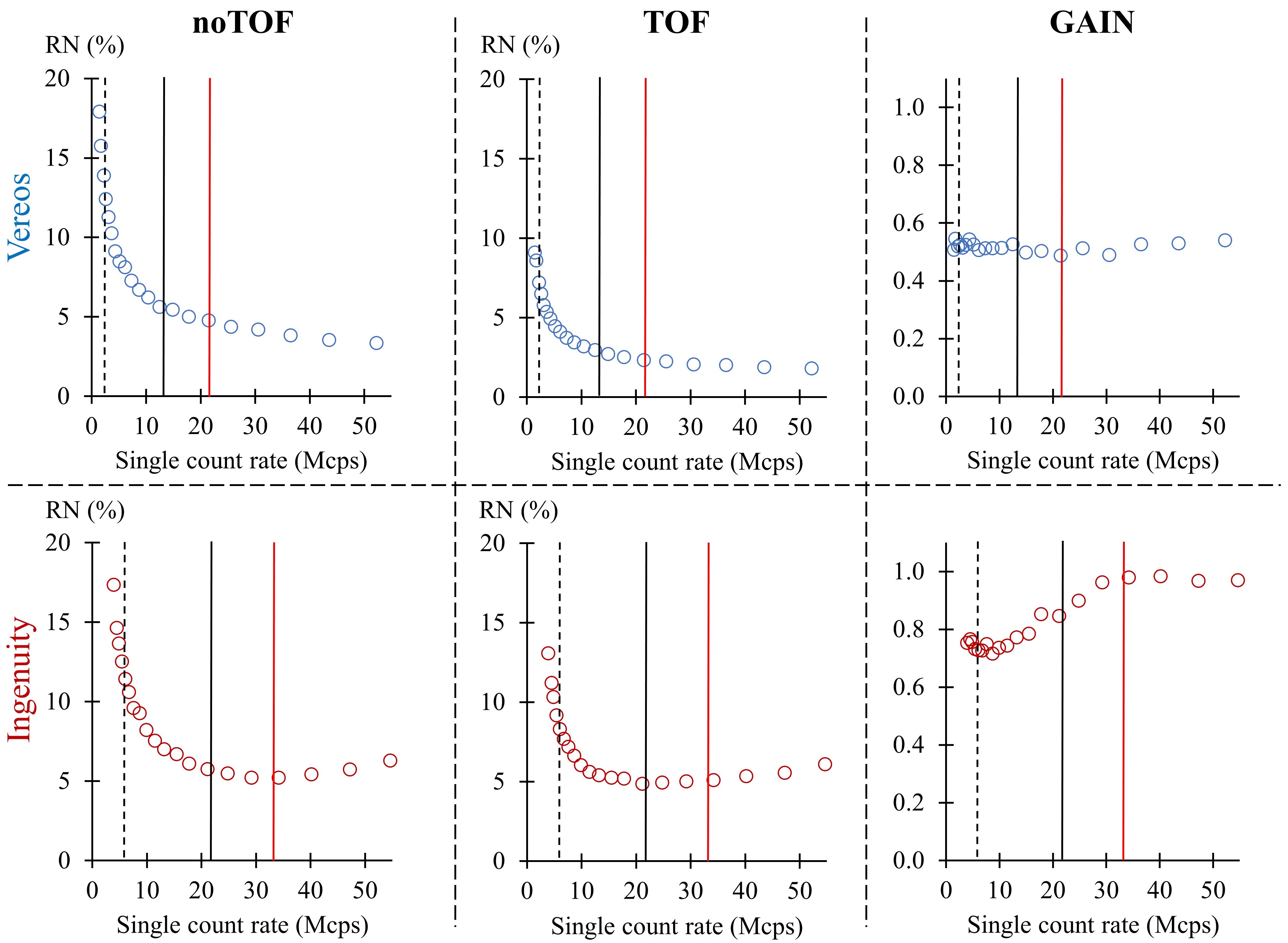

Supplement: Supplementary file 2 — Additional file 2: Figure S7. Single count rates versus background relative noise of the IEC phantom for the noTOF (left panels) and TOF (median panels) images from the Vereos (blue) and Ingenuity (red) cameras, together with the corresponding TOF-related gain, i.e. the ratio between the background relative noise of TOF and noTOF images (right panels). The vertical lines are defined in the legend of Figure 4. [file 40658_2020_344_MOESM2_ESM.jpg]

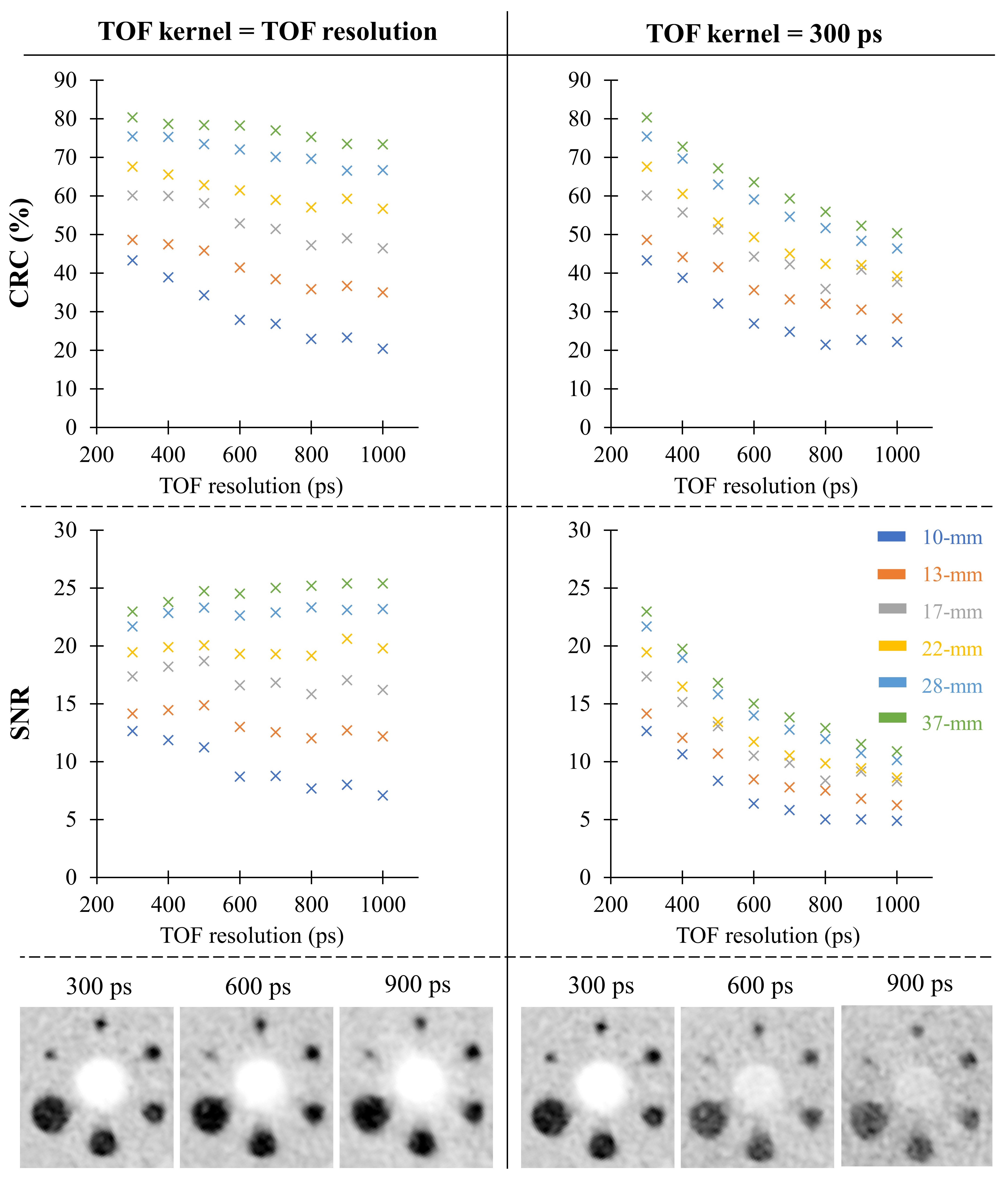

Supplement: Supplementary file 4 — Additional file 4: Figure S8. Data derived from simulated images of the IEC phantom, obtained with the GATE/Geant4 platform, reconstructed with the CASToR software (OSEM, 2 iterations 10 subsets) and confirming that incorrect TOF-kernel leads to a decrease in the contrast and SNR of TOF images. More precisely, these data show the comparative evolutions of the contrast recovery coefficients (upper panel) and SNR (middle panel) of hot spheres for increasing values of TOF resolution between the TOF images reconstructed with TOF-kernels matching exactly the increasing values of TOF resolution (left panel) and those reconstructed with misfit TOF-kernels kept unchanged at 300 ps (right panel). Simulated PET slices, obtained for various TOF resolutions, are shown in the bottom panels. [file 40658_2020_344_MOESM4_ESM.jpg]
