# Supplementary material for: Fully digital PET is unaffected by any deterioration in TOF resolution and TOF image quality in the wide range of routine PET count rates
Source: EJNMMI Phys. 2021 Jan 6;8:1. doi: 10.1186/s40658-020-00344-5 (PMC7788141; doi:10.1186/s40658-020-00344-5)
Supplement: Supplementary file 3 — Additional file 3: Table S3: Characteristics of currently commercialized PMT- and SiPM-based PET cameras with a special focus on the size of their trigger domains. [file 40658_2020_344_MOESM3_ESM.docx]

**Supplemental Table 3:** Characteristics of currently commercialized PMT- and SiPM-based PET cameras with a special focus on the size of their trigger domains.

|  | **PMT-based PET** | | | **SiPM-based PET** | | |
| --- | --- | --- | --- | --- | --- | --- |
| **Camera type** | **Ingenuity TF** | **Biograph mCT Flow** | **Discovery MI DR**  **4 rings** | **Vereos** | **Biograph Vision** | **Discovery MI**  **5 rings** |
| Reference | (*29*) | (*44*) | (*46*) | (*1*) | (*2*) | (*3*) |
| Scintillator | LYSO | LSO | LYSO | LYSO | LSO | LYSO |
| Photodetector | PMT | PMT | PMT | dSiPM | aSiPM | aSiPM |
| Number of scintillators | 29568 | 32448 | 13824 | 23040 | 60080 | 24480 |
| Number of photodetectors | 420 | 768 | 1024 | 23040 | 38912 | 12240 |
| Scintillator-to-photodetector coupling | 70:1 | 42:1 | 14:1 | 1:1 | 1.6:1 | 2:1 |
| **Trigger domain (cm^2^)** | **132.5** | **27.0** | **14.3** | **0.64** | **10.2** | **7.7** |
| Crystal dimension  (Trans x Ax x width mm^3^) | 4x4x22 | 4x4x20 | 4.2x 6.3x25 | 4x4x19 | 3.2x3.2x20 | 3.9x 5.3x25 |
| Ring diameter (mm) | 903 | 842 | 700 | 764 | 820 | 700 |
| Axial FOV (mm) | 180 | 221 | 157 | 164 | 263 | 250 |
| Energy window (keV) | 275.94 [439 – 715] | 215 [435 – 650] | 225  [425-650] | 163.52 [450–613] | 150 [435-585] | 225 [425-650] |

Ax: axial

Trans: transverse

FOV: field of view
