# Supplementary material for: Fully digital PET is unaffected by any deterioration in TOF resolution and TOF image quality in the wide range of routine PET count rates
Source: EJNMMI Phys. 2021 Jan 6;8:1. doi: 10.1186/s40658-020-00344-5 (PMC7788141; doi:10.1186/s40658-020-00344-5)
Supplement: Supplementary file 5 — Additional file 5: Appendix 1 IEC phantom assessment of our methodology of count rate estimation in patients [file 40658_2020_344_MOESM5_ESM.docx]

**Appendix 1: IEC phantom assessment of our methodology of count rate estimation in patients**

*Estimation of the single event rates associated with higher activities than those administered in our patients*

The extrapolation process was evaluated with the IEC phantom data recorded on the Vereos camera (see description of this experiment on the IEC phantom in Materials and Methods). For this purpose, the single event rate measured on the last consecutive recordings of the IEC phantom (i.e. that with the lowest activity concentration: $S_{last}^{Ver}$) was used for estimating the count rates corresponding to each of the other recordings (i.e. the $i$ recordings with higher activity concentrations than $S_{last}^{Ver}$ : $S_{estimated,i}^{Ver}$), by using the following equation:

$$S_{estimated,i}^{Ver}=f_{A\to S}^{Ver}\left[ C_{i}.f_{S\to A}^{Ver}(S_{last}^{Ver}) \right]$$

where $C_{i}$ corresponds to the ratio of activity concentration between the last recording and the considered $i$ recording, and functions $f_{S\to A}^{Ver}$ and $f_{A\to S}^{Ver}$ are described in the legend of Figure 1.

As shown in the Figure bellow, the relative difference between the actual measured single rates (i.e. those extracted from the list-mode data for each recording $i$ : $S_{measured,i}^{Ver}$) and the single rates extrapolated with this method ($S_{estimated,i}^{Ver}$) was no greater than 2% for all recordings.


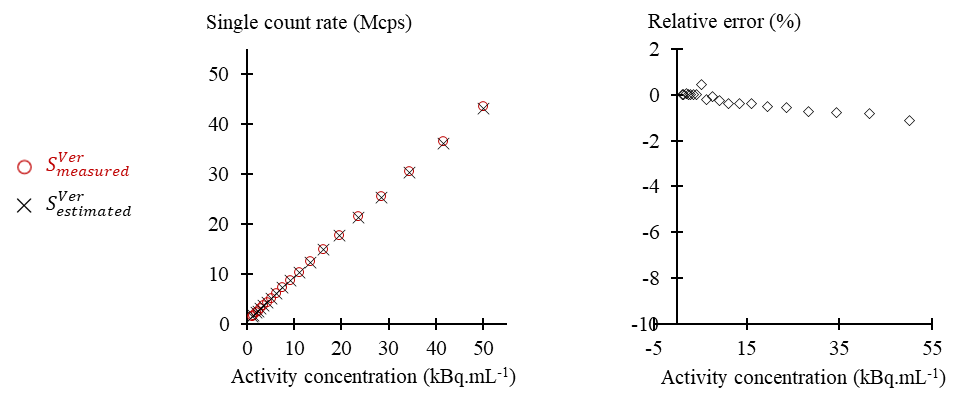


*Estimation of the count rates of the Ingenuity from those documented in patients with the Vereos camera*

The extrapolation process was assessed with the IEC phantom data recorded on the Vereos and Ingenuity cameras. For this purpose, the single event rate measured on the last consecutive recordings of the IEC phantom with the Vereos camera (i.e. that with the lowest activity concentration : $S_{last}^{Ver}$) was used for estimating the count rates corresponding to each of the $i$ recordings from the Ingenuity ($S_{estimated,i}^{Ing}$), by using the following equation:

$$S_{estimated,i}^{Ing}=f_{A\to S}^{Ing}\left[ C_{Ver\to Ing,i}.f_{S\to A}^{Ver}(S_{last}^{Ver}) \right]$$

where $C_{Ver\to Ing,i}$ corresponds to the ratio of activity concentration between the last Vereos recording and the considered Ingenuity recording $i$, and functions $f_{S\to A}^{Ver}$ and $f_{A\to S}^{Ing}$ are described in the legend of Figure 1.

As shown in the Figure bellow, the relative difference between the actual measured single rates (i.e. those extracted from the list-mode data for each recording $i$ ; $S_{measured,i}^{Ing}$) and the single rates extrapolated with this method ($S_{estimated,i}^{Ing}$) was no greater than 7% for all recordings.

**
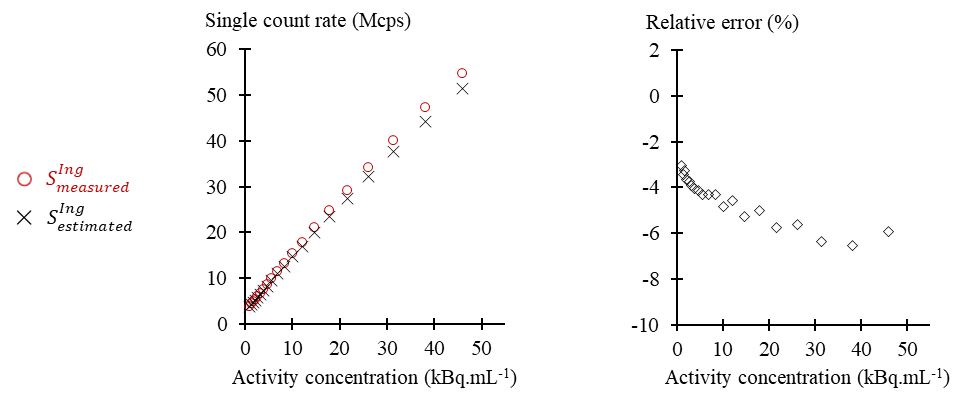
**
